# Supplementary figures and images for: The cooperative binding of TDP-43 to GU-rich RNA repeats antagonizes TDP-43 aggregation
Source: eLife. 2021 Sep 7;10:e67605. doi: 10.7554/eLife.67605 (PMC8523171; doi:10.7554/eLife.67605)

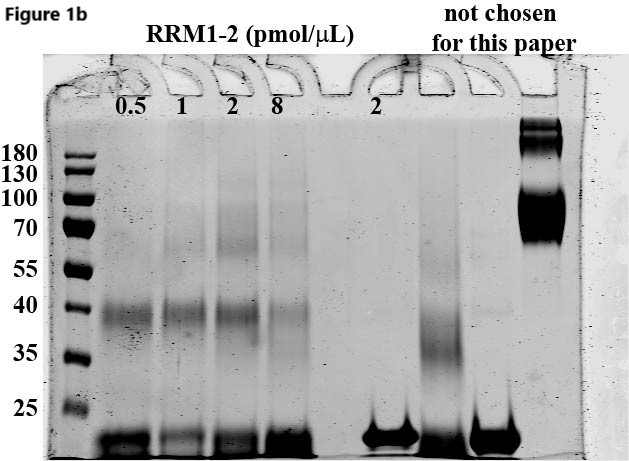

Supplement: Figure 1—source data 1. [file elife-67605-fig1-data1.zip › Fig 1b. Without Benzonase.(1).jpg]

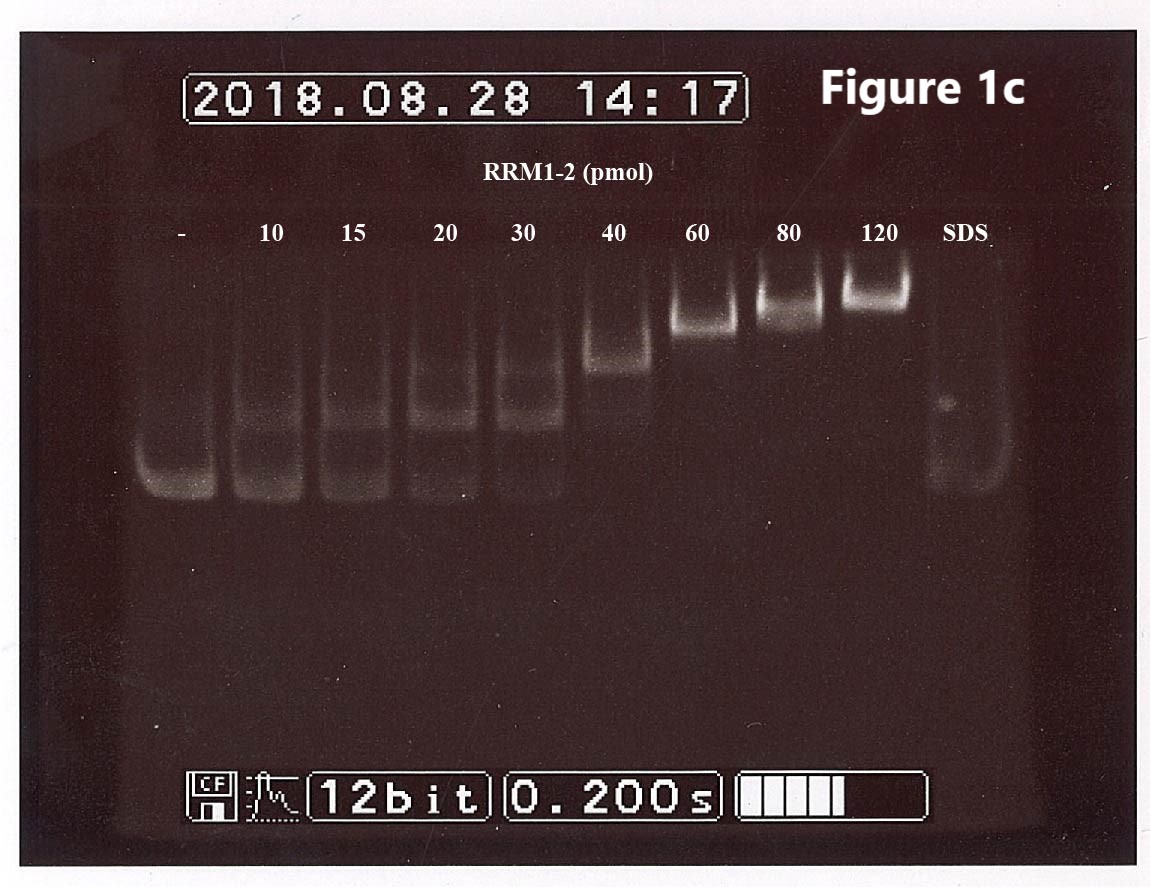

Supplement: Figure 1—source data 2. [file elife-67605-fig1-data2.zip › Figure 1c-source data 1 (RRM1-2).jpg]

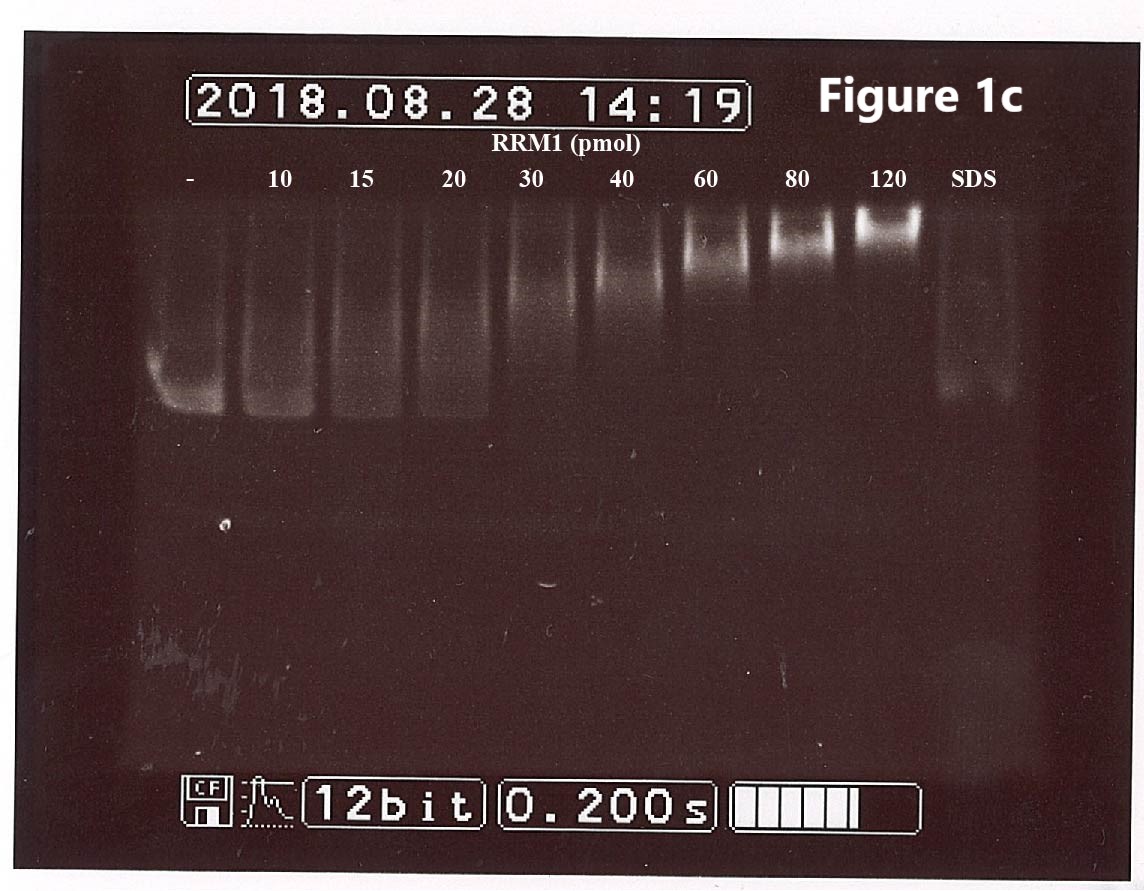

Supplement: Figure 1—source data 3. [file elife-67605-fig1-data3.zip › Figure 1c-source data 2 (RRM1).jpg]

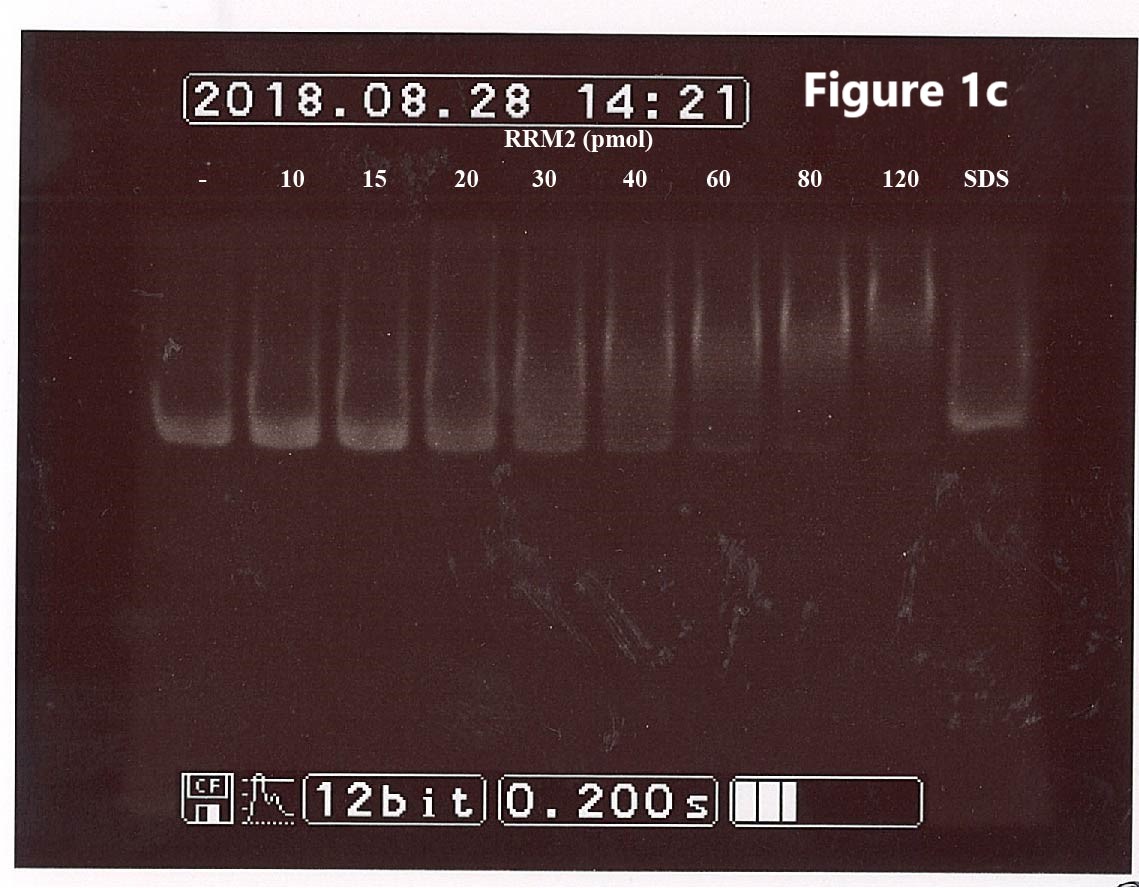

Supplement: Figure 1—source data 4. [file elife-67605-fig1-data4.zip › Figure 1c-source data 3 (RRM2).jpg]

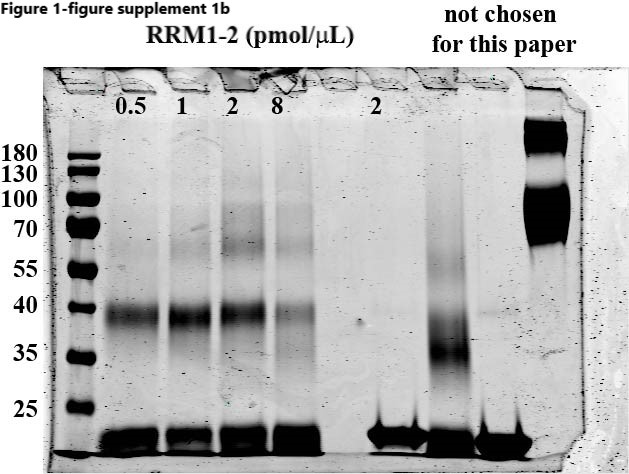

Supplement: Figure 1—figure supplement 1—source data 2. [file elife-67605-fig1-figsupp1-data2.zip › Fig S1b With Benzonase..jpg]

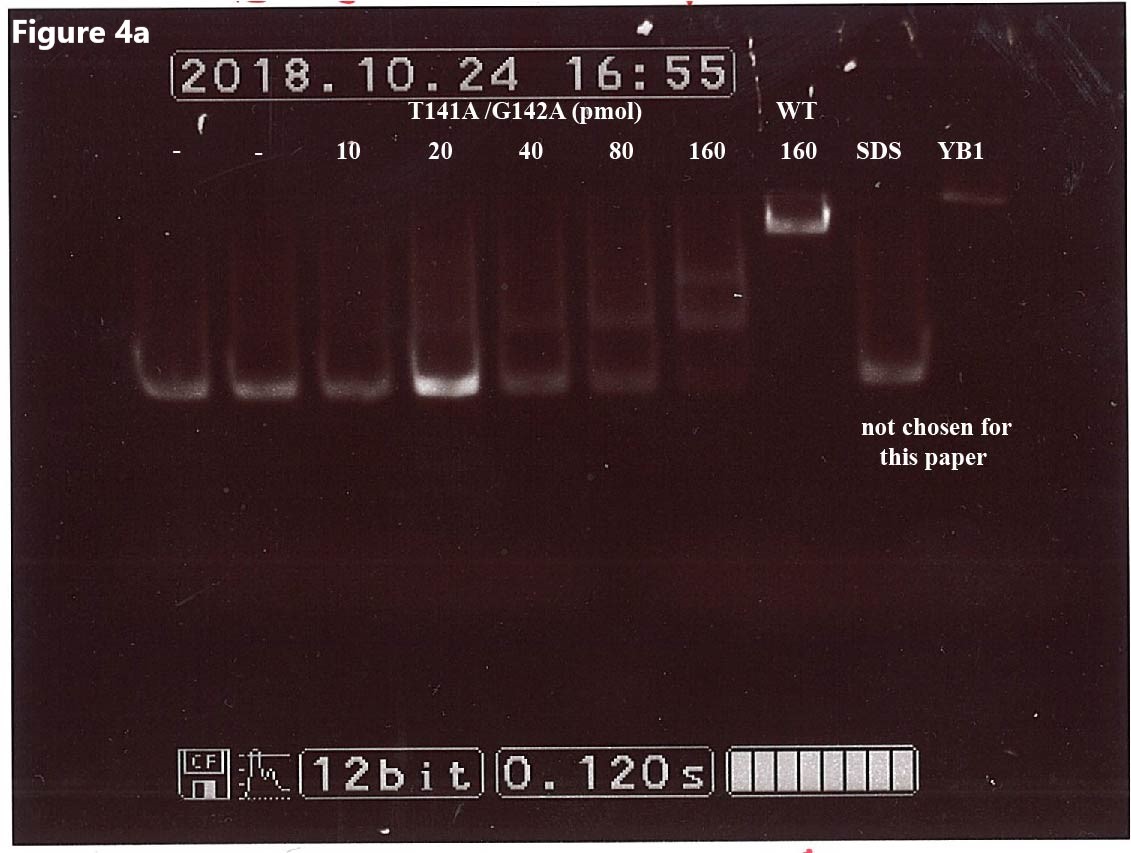

Supplement: Figure 4—source data 1. [file elife-67605-fig4-data1.zip › Figure 4a-source data 1 (T141A G142A)(3).jpg]

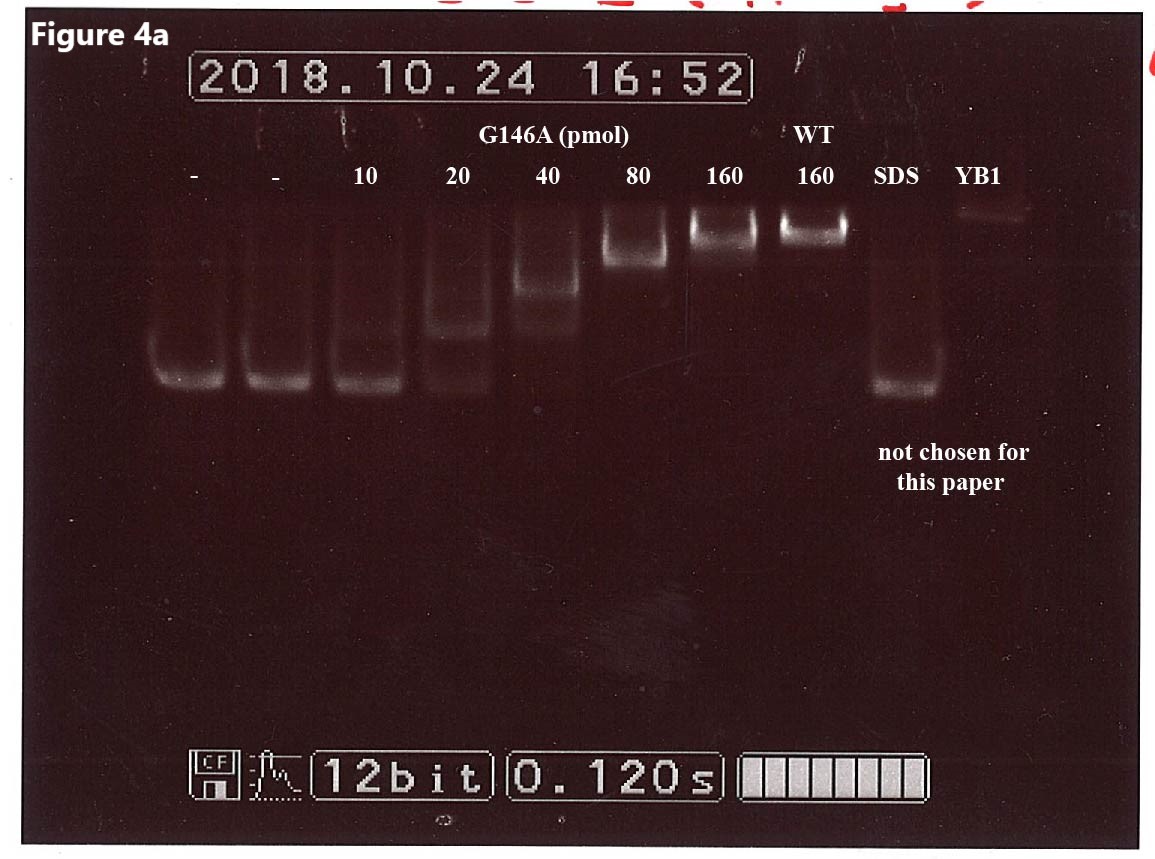

Supplement: Figure 4—source data 2. [file elife-67605-fig4-data2.zip › Figure 4a-source data 2 (G146A).jpg]

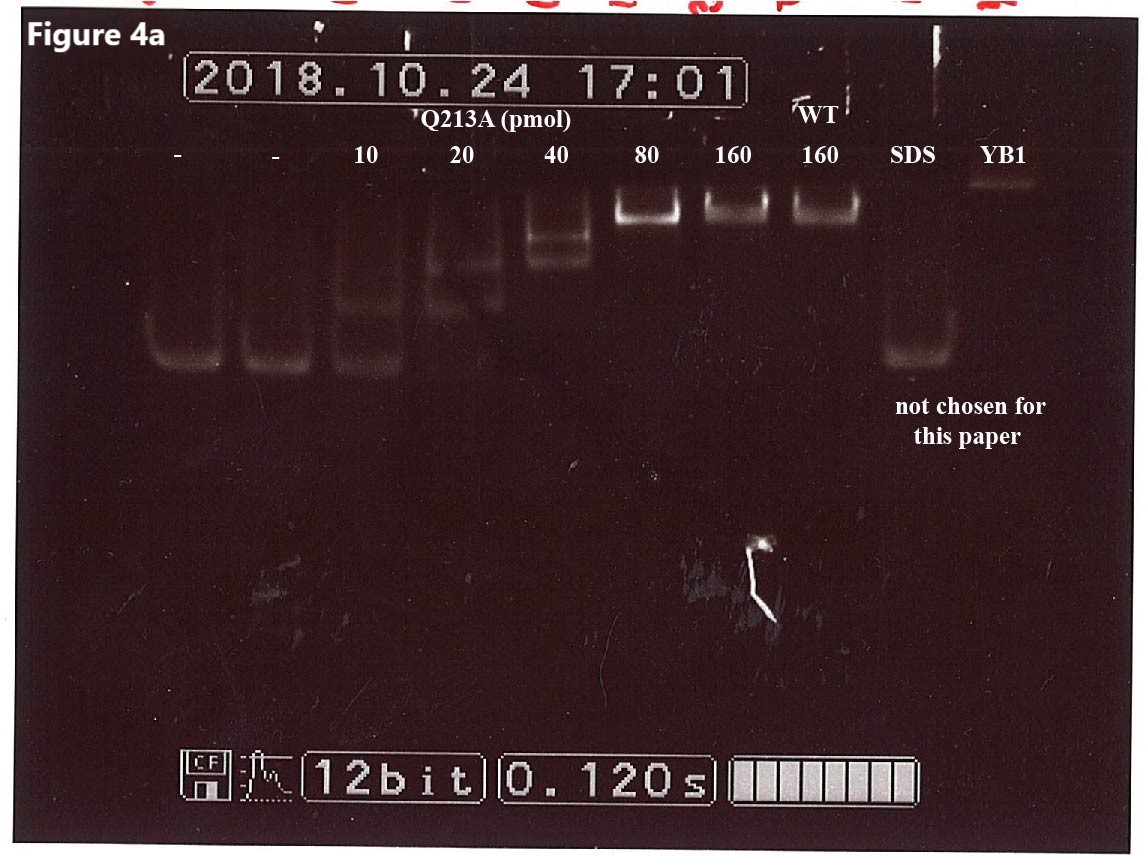

Supplement: Figure 4—source data 3. [file elife-67605-fig4-data3.zip › Figure 4a-source data 3 (Q213A).jpg]
