# Supplementary material for: The cooperative binding of TDP-43 to GU-rich RNA repeats antagonizes TDP-43 aggregation
Source: eLife. 2021 Sep 7;10:e67605. doi: 10.7554/eLife.67605 (PMC8523171; doi:10.7554/eLife.67605)
Supplement: Supplementary file 1. — Characteristic dimensions, Rg and Dmax, and molar mass (MMcorrelation volume) were obtained from data analysis. The theoretical masses (MMsequence) were calculated from the amino acid sequence. [file elife-67605-supp1.docx]

**Supplementary file 1.** SAXS experimental conditions and deduced parameters from collected data. Characteristic dimensions, Rg and Dmax, and molar mass (MMcorrelation volume) were obtained from data analysis. The theoretical masses (MMsequence) were calculated from the amino acid sequence.

| **Data collection parameters** | | | | |
| --- | --- | --- | --- | --- |
| Instrument | SWING (SOLEIL) | | | |
| Detector | Eiger4M (Dectris) | | | |
| Beam geometry | 0.8 mm x 0.15 mm | | | |
| Wavelength [Å] | 1.0 | | | |
| q-range [Å^-1^] | 0.006 < *q* < 0.50 | | | |
| Absolute scaling | Comparison with scattering from pure H_2_O | | | |
| Exposure/dead time [s] | 0.99 (0.01) | | | |
| Loading sample concentration [µM] | RRM1-2: 116 | RRM1-2: 125 (GU)_3_: 150 | RRM1-2: 116 (GU)_6_:116 | RRM1-2: 116 (GU)_12_: 29 |
|  | | | | |
|  | **RRM1-2** | **RRM1-2/(GU)_3_** | **RRM1-2/(GU)_6_** | **RRM1-2/(GU)_12_** |
| **Guinier analysis** | | | | |
| *R_g_* Guinier [Å] | 21.3±0.1 | 21.9±0.1 | 20.2±0.1 | 29.8±0.4 |
| q*Rg*-range | 0.19-1.30 | 0.25-1.30 | 0.25-1.30 | 0.18-1.30 |
| **P(r) analysis** | | | | |
| *R_g_* p(r) [Å] | 21.9±0.1 | 22.3±0.1 | 20.3±0.1 | 30.7±0.2 |
| q-range [Å^-1^] | 0.010 - 0.40 | 0.010 - 0.40 | 0.010 - 0.40 | 0.010 - 0.40 |
| *D_Max_* [Å] | 80 | 80 | 65 | 105 |
| **Molecular mass determination** | | | |  |
| MM_sequence_ [kDa] | 20.7 | 22.8 | 24.8 | 49.4 |
| MM_correlation volume_  [kDa] | 20.6 | 21.6 | 24.1 | 47.0 |
