# Supplementary material for: The cooperative binding of TDP-43 to GU-rich RNA repeats antagonizes TDP-43 aggregation
Source: eLife. 2021 Sep 7;10:e67605. doi: 10.7554/eLife.67605 (PMC8523171; doi:10.7554/eLife.67605)
Supplement: Supplementary file 2. — Physical parameters of the established interactions between atoms of residues of RRM2 pocket around V220 and those located in RRM1 loop 3 from monomer 1 and 2, respectively, are shown. Values in brackets indicates the energy contribution (in kcal/mol) of amino acid residues to the protein-protein interface stability. Energies were averaged over 100 ns of MD simulation and values are reported in kcal/mol with variant of fluctuations being ±0.1 kcal/mol. 1bb and vdW correspond to backbone and van der Waals, respectively. [file elife-67605-supp2.docx]

Supplementary file 2. Bonds involved in the multimerization of TDP-43 as deduced from the complex RRM1-2/(GU)_12_ MD model. Physical parameters of the established interactions between atoms of residues of RRM2 pocket around V220 and those located in RRM1 loop 3 from monomer 1 and 2, respectively, are shown. Values in brackets indicates the energy contribution (in kcal/mol) of amino acid residues to the protein-protein interface stability. Energies were averaged over 100 ns of MD simulation and values are reported in kcal/mol with variant of fluctuations being ± 0.1 kcal/mol. *^1^bb and vdW correspond to backbone and van der Waals, respectively.*

| **Monomer 1** | **Atom** | **Monomer 2** | **Atom** | **Distance (**Å**)** | **Interaction type** | **Energy contribution** |
| --- | --- | --- | --- | --- | --- | --- |
|  |  |  |  |  |  |  |
| E204 | Oε2 | K140  (-6.21) | Hζ3 | 1.83 | ionic | -8.27 |
|  | Cδ |  | Cε | 3.7 | vdW*^1^* |  |
|  | Cδ | T141  (-2.06) | Cγ | 4.01 | vdW |  |
|  | Cγ |  | Cγ | 4.05 | vdW |  |
| D205 | Cβ | K140  (-2.14) | Cγ | 3.8 | vdW | -2.14 |
| R208 | HH12  (of NH1) | D138  (-0.72) | O (C=O) | 2.27 | H-bond | -4.9 |
|  | HH11  (of NH1) | L139  (-1.51) | O (C=O) | 2.42 | H-bond |  |
|  | HH12  (of NH1) |  | O (C=O) | 2.55 | H-bond |  |
|  | HH11  (of NH1) | K140  (-0.81) | O (C=O) | 2.29 | H-bond |  |
|  | HH11  (of NH1) | T141  (-1.47) | O (C=O) | 2.68 | H-bond |  |
|  | NH1 | G142 ( -0,4) | HN (bb*^1^*) | 3.43 | H-bond |  |
| M218 | O (C=O) | K137  (-2.85) | Nζ | 3.24 | H-bond | -2.85 |
| D219 | Oδ2 | K137  (-1.41) | Nζ | 4.26 | ionic | -7.22 |
|  | Cβ | T141  (-2.22) | Cγ2 | 3.1 | vdW |  |
|  | Cβ | G142  (-1.21) | Cα | 4.3 | vdW |  |
|  | Cγ | H143  (-2.38) | Cβ | 3.76 | vdW |  |
|  | Cβ |  | Cβ | 3.53 | vdW |  |
| V220 | HN (bb*^1^*) | T141  (-8.06) | O (C=O) | 1.82 | H-bond | -9.47 |
|  | O (C=O) |  | Hγ  (of Oγ) | 3.24 | H-bond |  |
|  | HN (bb) | H143  (-1.41) | Nδ1 | 3.5 | H-bond |  |
| F221 | Cβ | H143  (-0.67) | Cε1 | 3.08 | vdW | -0.67 |
