# Supplementary material for: The cooperative binding of TDP-43 to GU-rich RNA repeats antagonizes TDP-43 aggregation
Source: eLife. 2021 Sep 7;10:e67605. doi: 10.7554/eLife.67605 (PMC8523171; doi:10.7554/eLife.67605)
Supplement: Supplementary file 3. — a and b correspond to apparent dissociation constants KD1 and KD2, respectively. The thermodynamic parameters (ΔH, TΔS, ΔG) and χ2 values were expressed in kcal/mol and (kcal/mol)2, respectively. [file elife-67605-supp3.docx]

**Supplementary file 3:** Poly (GT) repeats binding capacity (N), apparent dissociation constant (*K*_D_), and thermodynamic parameters for RRM fragments of TDP-43, as determined by ITC. *^a^* and *^b^* correspond to apparent dissociation constants *K*_D1_ and *K*_D2_, respectively. The thermodynamic parameters (ΔH, TΔS, ΔG) and χ^2^ values were expressed in kcal/mol and (kcal/mol)^2^, respectively.

| **Protein** | **Poly (GT) repeats** | **N** | **K_D_ (M)** | **ΔH** | **TΔS** | **ΔG** | **χ^2^** |
| --- | --- | --- | --- | --- | --- | --- | --- |
| **RRM1-2** | **(GT)_3_** | 1.70 ± 2.4e^-2^ | 2.2e^-6^ ± 177e^-9^ | -16.7 ± 0.413 | -9.00 | -7.71 | 0.036 |
|  | **(GT)_6_** | 0.99 ± 4.1e^-3^ | 68.4e^-9^ ± 8.68e^-9^ | -29.8 ± 0.345 | -20.00 | -9.78 | 0.509 |
|  | **(GT)_12_** | 0.40 ± 1.9e^-3^ | **^a^** 51.0e^-9^ ± 1.20e^-10^  **^b^** 0.4e^-9^ ± 5.34e^-12^ | -14.8 ± 0.781  -76.4 ± 0.785 | -4.86  -63.60 | -9.95  -12.80 | 2.14 |
| **RRM1** | **(GT)_3_** | 0.82 ± 0.6e^-3^ | 929.0e^-9^ ± 56.2e^-9^ | -18.5 ± 0.262 | -10.20 | -8.23 | 0.040 |
|  | **(GT)_6_** | 0.39 ± 6.2e^-4^ | **^a^** 84.4e^-9^ ± 1.81e^-10^  **^b^** 9.4e^-9^ ± 7.87e^-11^ | -9.9 ± 0.163  -40.4 ± 0.182 | -0.23  -29.50 | -9.65  -10.90 | 0.075 |
|  | **(GT)_12_** | 0.24 ± 1.2e^-3^ | **^a^** 2.3e^-6^ ± 2.31e^-6^  **^b^** 54.2e^-9^ ± 4.11e^-10^ | -14.7 ± 0.569  -78.0 ± 0.321 | -7.03  -68.10 | -7.69  -9.91 | 0.391 |
| **RRM2** | **(GT)_3_** | 0.76 ± 6.3e^-3^ | 679.0e^-9^ ± 47.4e^-9^ | -16.5 ± 0.250 | -8.04 | -8.42 | 0.046 |
|  | **(GT)_6_** | 0.30 ± 4.2e^-3^ | **^a^** 31.6e^-9^ ± 2.56e^-9^  **^b^** 601e^-9^ ± 6.46e^-9^ | -28.8 ± 0.202  -15.9 ± 0.191 | -18.60  -7.38 | -10.20  -8.49 | 0.021 |
|  | **(GT)_12_** | 0.24 ± 4.6e^-3^ | **^a^** 347.0e^-9^ ± 8.58e^-9^  **^b^** 5.28e^-6^ ± 45.5e^-9^ | -79.9 ± 1.140  -0.17 ± 0.637 | -71.10  -7.03 | -8.81  -7.20 | 0.505 |
