# Supplementary material for: The cooperative binding of TDP-43 to GU-rich RNA repeats antagonizes TDP-43 aggregation
Source: eLife. 2021 Sep 7;10:e67605. doi: 10.7554/eLife.67605 (PMC8523171; doi:10.7554/eLife.67605)
Supplement: Supplementary file 4. — a and b correspond to apparent dissociation constants KD1 and KD2, respectively. The thermodynamic parameters (ΔH, TΔS, ΔG) and χ2 values were expressed in kcal/mol and (kcal/mol)2, respectively. [file elife-67605-supp4.docx]

**Supplementary file 4:** Poly (GT) repeats binding capacity (N), apparent dissociation constant (*K*_D_), and thermodynamic parameters for RRM1-2 protein mutants, as determined by ITC. *^a^* and *^b^* correspond to apparent dissociation constants *K*_D1_ and *K*_D2_, respectively. The thermodynamic parameters (ΔH, TΔS, ΔG) and χ^2^ values were expressed in kcal/mol and (kcal/mol)^2^, respectively.

| **Protein** | **Poly (GT) repeats** | **N** | **K_D_ (M)** | **ΔH** | **TΔS** | **ΔG** | **χ^2^** |
| --- | --- | --- | --- | --- | --- | --- | --- |
| **T141A/G142A** | **(GT)_6_** | 0.89 ± 2.9e^-3^ | 50.9e^-9^ ± 3.12e^-9^ | -30.3 ± 0.182 | -20.40 | -9.95 | 0.111 |
|  | **(GT)_12_** | 0.40 ± 1.1e^-4^ | **^a^** 52.3e^-9^ ± 4.23e^-10^  **^b^** 3.8e^-9^ ± 7.47e^-11^ | -15.3 ± 0.441  -63.1 ± 0.345 | -5.33  -51.60 | -9.93  -11.50 | 0.264 |
| **G146A** | **(GT)_6_** | 0.82 ± 2.1e^-3^ | 77.9e^-9^ ± 5.01e^-9^ | -26.0 ± 0.204 | -16.30 | -9.70 | 0.084 |
|  | **(GT)_12_** | 0.41 ± 4.3e^-3^ | **^a^** 196e^-9^ ± 4.83e^-10^  **^b^** 10.1e^-9^ ± 6.81e^-11^ | -17.0 ± 0.321  -72.3 ± 0.253 | -7.88  -61.40 | -9.15  -10.90 | 0.252 |
| **Q213A** | **(GT)_6_** | 0.83 ± 4.3e^-3^ | 48.3e^-9^ ± 7.86e^-9^ | -31.4 ± 0.458 | -21.40 | -9.98 | 0.799 |
|  | **(GT)_12_** | 0.36 ± 1.0e^-3^ | **^a^** 50.4e^-9^ ± 3.02e^-10^  **^b^** 0.2e^-9^ ± 6.39e^-12^ | -16.1 ± 0.409  -81.2 ± 0.435 | -6.18  -67.80 | -9.96  -13.40 | 0.626 |
