# Supplementary material for: The cooperative binding of TDP-43 to GU-rich RNA repeats antagonizes TDP-43 aggregation
Source: eLife. 2021 Sep 7;10:e67605. doi: 10.7554/eLife.67605 (PMC8523171; doi:10.7554/eLife.67605)
Supplement: Supplementary file 5. [file elife-67605-supp5.pdf]

Eurofins Genomics Europe Applied Genomics GmbH, Anzinger Str. 7 a, D-85560 Ebersberg

Vandana Joshi
UNIVERSITE EVRY VAL
D'ESSONNE,
SABNP/INSERM1204
Rue Père André Jarlan 91025 EVRY cedex.
91000, Évry
France

SOP_APG_Zelllinienauthentizität_A04_1.0

Report date: 11.08.2021

Certificate
Cell Line Authentication Test
Order ID: 11107530406

Method:
DNA isolation was carried out from cell pellet (cell layer).
Genetic characteristics were determined by PCR-single-locus-technology.
16 independent PCR-systems D8S1179, D21S11, D7S820, CSF1PO, D3S1358, TH01, D13S317, D16S539, D2S1338, AMEL, D5S818, FGA, D19S433, vWA, TPOX and D18S51 were investigated.
(ASN-0002 core markers are colored grey, Thermo Fisher, AmpFISTR® Identifiler® Plus PCR Amplification Kit)
In parallel, positive and negative controls were carried out yielding correct results.

Result:

**Client Sample Name**	Hela-1	Hela-2	Hela-3
**Sample Code**	CL00005845	CL00005846	CL00005847
D8S1179	12,13	12,13	12,13
D21S11	27,28	27,28	27,28
D7S820	8,12	8,12	8,12
CSF1PO	9,10	9,10	9,10
D3S1358	15,18	15,18	15,18
TH01	7,7	7,7	7,7
D13S317	12,13.3	12,13.3	12,13.3
D16S539	9,10	9,10	9,10
D2S1338	17,17	17,17	17,17
D19S433	13,14	13,14	13,14
vWA	16,18	16,18	16,18
TPOX	8,12	8,12	8,12
D18S51	16,16	16,16	16,16
AMEL	X,X	X,X	X,X
D5S818	11,12	11,12	11,12
FGA	21,21	21,21	21,21
**Database Name**	HELA	HELA	HELA

The table shows the result of the cell line analysis and the comparison with the online database of the DSMZ (http://www.dsmz.de/de/service/services-human-and-animal-cell) and the Cellosaurus database (https://web.expasy.org/cellosaurus). Please note that only the PCR-systems according to ANSI/ATCC standard ASN-0002 were aligned (D5S818, D13S317, D7S820, D16S539, VWA, TH01, TPOX, CSF1PO, AMEL - colored grey).

This report was created automatically and is therefore valid without a signature.
